# Cancer Screening in the Coronavirus Pandemic Era: Adjusting to a New Situation

**DOI:** 10.1200/GO.21.00033

**Published:** 2021-03-30

**Authors:** Partha Basu, Samar Alhomoud, Katayoun Taghavi, Andre L. Carvalho, Eric Lucas, Iacopo Baussano

**Affiliations:** ^1^ International Agency for Research on Cancer, Lyon, France; ^2^ Section Colorectal Surgery, King Faisal Specialist Hospital & Research Center, Riyadh, Saudi Arabia; ^3^ Institute of Social and Preventive Medicine, University of Bern, Bern, Switzerland; ^4^ The Graduate School for Cellular and Biomedical Sciences (GCB) of the University of Bern, Bern, Switzerland

## Abstract

**PURPOSE:**

The coronavirus-induced pandemic has put great pressure on health systems worldwide. Nonemergency health services, such as cancer screening, have been scaled down or withheld as a result of travel restrictions and resources being redirected to manage the pandemic. The present article discusses the challenges to cancer screening implementation in the pandemic environment, suggesting ways to optimize services for breast, cervical, and colorectal cancer screening.

**METHODS:**

The manuscript was drafted by a team of public health specialists with expertise in implementation and monitoring of cancer screening. A scoping review of literature revealed the lack of comprehensive guidance on continuation of cancer screening in the midst of waxing and waning of infection. The recommendations in the present article were based on the advisories issued by different health agencies and professional bodies and the authors' understanding of the best practices to maintain quality-assured cancer screening.

**RESULTS:**

A well-coordinated approach is required to ensure that essential health services such as cancer management are maintained and elective services are not threatened, especially because of resource constraints. In the context of cancer screening, a few changes in invitation strategies, screening and management protocols and program governance need to be considered to fit into the new normal situation. Restoring public trust in providing efficient and safe services should be one of the key mandates for screening program reorganization. This may be a good opportunity to introduce innovations (eg, telehealth) and consider de-implementing non–evidence-based practices. It is necessary to consider increased spending on primary health care and incorporating screening services in basic health package.

**CONCLUSION:**

The article provides guidance on reorganization of screening policies, governance, implementation, and program monitoring.

**CONTEXT**

**Key Objective**
What are the challenges at health system level for reinitiating cancer screening in the postpandemic scenario, and how to optimize screening services by making them resilient to similar contextual threats?
**Knowledge Generated**
Restarting cancer screening activities in the post-COVID era will require a well-coordinated effort to reach out to the community more proactively, alleviate the concerns of the apparently healthy individuals to return to routine health care and reorganize clinical services to minimize backlogs in services. This should be considered as an opportunity to improve organization, quality, and reach of the cancer screening programs through pragmatic application of technology and innovations and de-implementation of existing non–evidence-based practices.
**Relevance**
The best practices in governance, organization, and implementation highlighted in the article will help the cancer screening programs to encounter disruptive consequences of the pandemic better and thus prevent the predicted surge in the number of deaths from cancers, especially in limited-resourced countries.

## INTRODUCTION

The SARS-CoV-2 infection responsible for the first pandemic of the twenty-first century already claimed more than 2 million lives as of January 2021.^1^ Health system in every country is strained to the extreme as a result of the impact of the COVID-19 pandemic. Services deemed nonemergency such as cancer screening were scaled down or stopped as part of efforts to reduce risks of SARS-CoV-2 infection and also to reduce load on the health services. Measures to avoid nonurgent interactions with health facilities were endorsed by the WHO and the respective Ministry of Health during the acute phase of the transmission in the community.^2^ Different professional medical societies and voluntary organizations also advised to put cancer screening on hold.^3,4^ Most importantly, the screen-eligible individuals were hesitant to visit the health facilities because of the scare of getting the infection. As a consequence, a significant surge in the number of deaths from cancer and other diseases unrelated to COVID-19 is predicted in the near future, especially in the socioeconomically disadvantaged and other vulnerable populations.^5^

As the countries ease restrictions and reopen various essential health care facilities, putting cancer screening and management back on track will continue to face challenges. A survey conducted by the WHO in May 2020 in 155 countries not only reported major disruption of noncommunicable disease (NCD) control services (including cancer screening) in almost all countries but also highlighted the difficulties of reinitiating such activities.^2^ Full or partial assignment of the dedicated health staff for NCD control to support COVID-19 in 94% of the responding countries is one such example. Several countries have already reported more than 90% drop in screening, diagnostic, and treatment activities following the declaration of a health emergency because of COVID-19.^6-8^ Scotland reported a 70% reduction in urgent referrals of patients with suspected cancer by primary care physicians during the surge of COVID-19 cases, and such drastic reduction in referrals will significantly delay cancer detection.^9,10^

The public health policies and social measures adopted by the countries to respond to the pandemic are different, and there is no one size fits all solution to reorient health service components and maintain the nonemergency preventive services. Restarting cancer screening activities as the crisis situation continues or somewhat settles down will require a well-coordinated effort to reach out to the community more proactively, alleviate the concerns of the apparently healthy individuals to return to routine health care and reorganize clinical services to minimize backlogs in services.

The objectives of this article are to enumerate the challenges at health system level for reinitiating cancer screening services in the post-COVID scenario and to suggest various approaches to optimize services for breast, cervical, and colorectal cancer (CRC) screening and improve their resilience to contextual threats.

## COVID PANDEMIC—DIFFERENT SCENARIOS AND THEIR LIKELY IMPACT ON PUBLIC HEALTH INTERACTIONS

**FIG 1:**
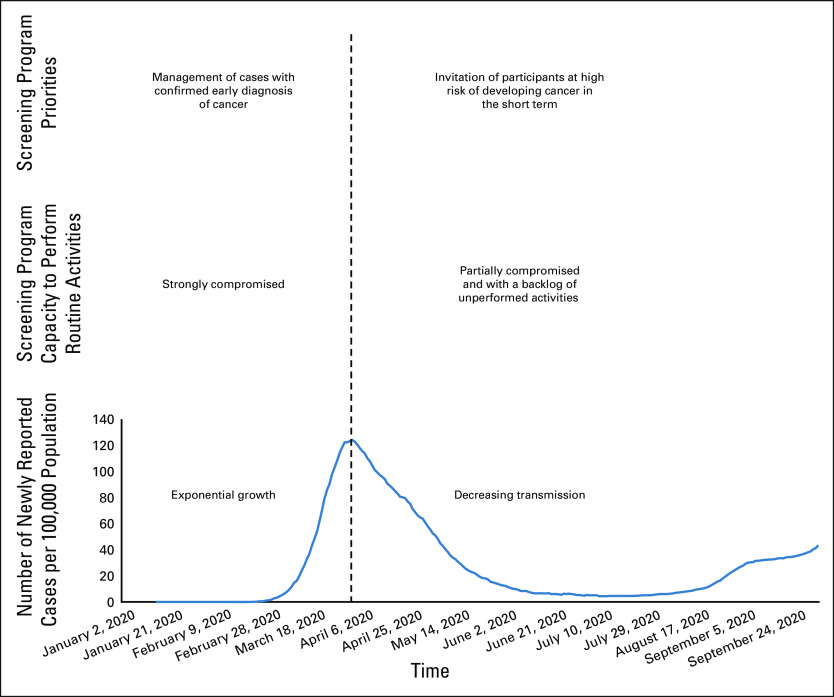
Illustration of the impact on cancer screening activities of the COVID-19 epidemic phases.

Characteristically, the unfolding of the COVID-19 epidemic occurs through a sequence of phases with different levels of infection transmission and burden of disease in the population (Fig 1). In the first phase, when effective mitigation measures are not yet in place, the number of cases grows exponentially, and health care systems may get overwhelmed with management of the symptomatic cases. Consequently, routine activities are severely disrupted and most nonurgent medical activities are temporarily suspended. In the second phase, if public health control measures are effective, infection transmission slows down and the number of new infections decreases. In the absence of an effective vaccine, several waves of exponential growth may occur, until a sufficient fraction of the population has developed protective immunity against the infection, at this point the epidemic declines and enters in the extinction phase (not yet observed with SARS-CoV-2).

The stringency of public health control measures and restrictions is modulated according to the intensity of each phase of the epidemic; the ability of health care facilities to deal with routine activities is affected correspondingly. In very acute phases of the epidemic, available human and technologic resources are mostly reallocated from routine health care activities to COVID-19 response. Subsequently, during less acute phases of the epidemic, the health care system not only resumes routine actions with limited resources and workforce but also faces a substantial backlog of activities. In the context of screening programs, the efficiency of the program has to be significantly augmented within a short period to cope with these pending residual activities. Of note, program's efficiency will be further negatively affected by logistical measures put in place to protect screening participants from the risk of SARS-CoV-2 transmission.

## POLICIES, GOVERNANCE, AND COORDINATION OF CANCER SCREENING

Ministries of Health should be alerted to the fact that cancer is an ongoing pandemic, which is fast approaching the milestone of claiming nearly 10 million lives every year.^12^ Undeniably, decision making and balancing the demand of so many competing health priorities even as the SARS-CoV-2 outbreak moves to a less acute phase will be extremely challenging for policy makers. There is a possibility that economic slowdown and reallocation of public health resources to pandemic response will be put forward as a justification to reduce funding for NCD control. A well-coordinated approach is required to ensure that essential health services are maintained and routine and elective services are not threatened, especially because of resource constraints, fostering a sense of public trust. Hospitals or clinics aiming to initiate cancer screening and other essential health services should create COVID-protected areas segregated from the movement of known or suspected COVID individuals. These protected areas should be routinely cleaned and decontaminated, and both staff and screening attendees should follow the norms of personal protection.

Until a significant proportion of the population is vaccinated with any of the available vaccines against SARS-CoV-2, the course of the outbreak is likely to wax and wane, and the strategic responses need to be dynamic and in sync with the situation. There are governance and coordination mechanisms that might help to optimize cancer screening and downstream activities, and these are listed in Table 1. One of the key requirements would be to increase spending on primary health care by at least 1% of the GDP, which according to the WHO is within the capacity of even the low-income countries.^13^

**TABLE 1:**
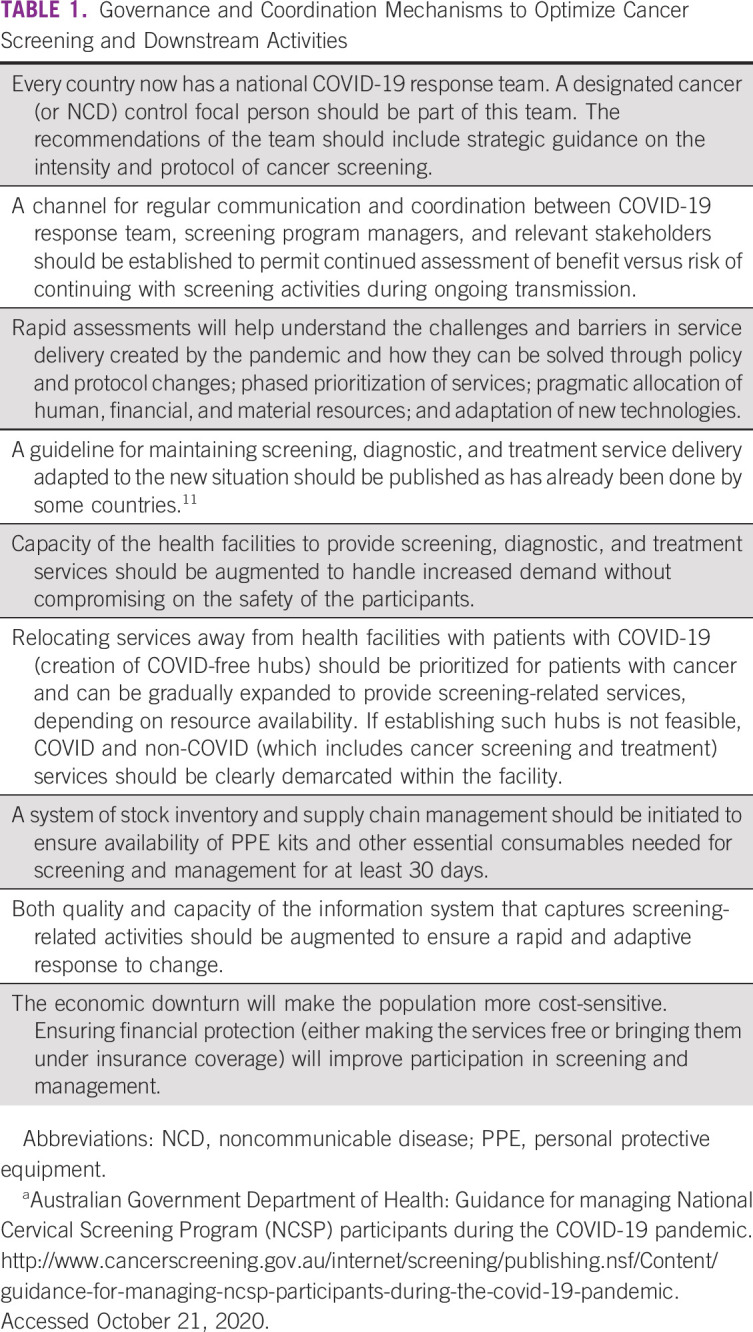
Governance and Coordination Mechanisms to Optimize Cancer Screening and Downstream Activities

There are still uncertainties around the appropriate policy to return health care workers to service or permitting nonemergency attendance of patients to the hospitals after a positive SARS-CoV-2 test. Although most health facilities would recommend a negative test obtained after the resolution of symptoms, some institutions might exclude health care workers and patients until a fixed period of time since symptom recovery. Four weeks may be a safer interval between symptom onset and nonemergency contact with health facilities when real-time reverse transcription polymerase chain reaction cannot be repeated.^14^

## COMMUNITY MOBILIZATION AND EFFECTIVE COMMUNICATION STRATEGIES

The infodemic associated with COVID-19 has generated a lot of myths and misconceptions that are likely to have a lasting impact on cancer screening services. It is important to understand the concerns of the screen-eligible individuals through sample surveys or qualitative research, which will help the program restructure strategies for invitation and community mobilization. Clear messaging from all stakeholders on the need to continue cancer screening and publicizing the robustness of the safety measures at the health facilities associated with screening would create public trust and improve their care-seeking behavior. Major policy changes (eg, reopening of cancer screening activities) should be widely publicized through mass media. Screening programs with systematic invitation strategies should inform the eligible populations through letters, emails, telephone calls, or digital platforms about the measures taken by the program to minimize the risk of SARS-CoV-2 transmission. The opportunistic programs should ensure that the health staff entrusted to mobilize individuals for screening are appropriately trained to provide such information in an unambiguous manner. In some countries (eg, Thailand or India), community health workers make home visits to counsel and invite individuals to cancer screening. Creative use of mobile phones and innovative apps may help the health workers to contact eligible individuals more efficiently while minimizing person-to-person contact. Cancer awareness campaigns should aim to improve public health literacy on the consequences of delaying screening, diagnosis, and treatment and involve various stakeholders, including the NGOs and faith-based organizations. Informing populations of the early symptoms of common cancers and the consequences of ignoring them because of the outbreaks should be a key component of all campaigns.

## MANAGING SCREENING INVITATIONS OR OFFERING SCREENING IN OPPORTUNISTIC SETTINGS

COVID-19 creates major disruptions in the systematic invitation procedure with screening appointments being delayed, deferred, or neglected during the acute phase of the pandemic. Strategies to deal with the backlog include increasing the number of screenings performed in a day (by extending the hours of service or by expanding the capacity), switching to more throughput testing technologies (eg, use of quantitative rather than qualitative fecal immunochemical tests (FITs) for CRC screening and introducing human papillomavirus [HPV] test for cervical cancer screening), and task-shifting or -sharing. Health systems should prioritize inviting those who are at highest risk (eg, the defaulters to screening, the post-treatment follow-ups, the women living with HIV for cervical cancer screening, etc). Individuals with known comorbidities should be given appointment in a way to minimize wait time at the health facilities, and some of them with least risk (eg, women with history of regular and normal cervical cancer screening nearing the upper age limit) may be spared of any further screening. Information system should be adapted and calibrated to be able to manage the new program strategies in appointment and invitation management. Helplines or dedicated websites may be set up to address patient queries.

Opportunistic screening is likely to decline significantly as the in-person visits by the asymptomatic patients to health facilities drop significantly during and after the acute situation.^15^ Primary care providers need to be more proactive in counseling and motivating the eligible populations. Remote consultation through telemedicine is becoming popular in many countries with improved technology and adoption of regulatory and legal framework.^16^ These telehealth opportunities or virtual clinics could be used to invite eligible men and women to screening. As a result of rationing of personal protective equipments (PPEs) in the face of supply shortage, screening appointments may be given on selected days of the week to examine a large number of participants on a single day.

## ADMINISTERING SCREENING TESTS AND MANAGING THE SCREEN-POSITIVES

Restoration of screening or diagnostic services should continue to mitigate the risk of SARS-CoV-2 transmission and at the same time have contingency plans anticipating the likelihood of restrictive measures being reintroduced. Reorganization of screening services as the COVID-19 case load reduces should focus on capacity enhancement as well as adopting novel approaches to minimize person-to-person contact and maintain social distancing. Written standard operating procedures for infection control should be circulated among the providers and displayed prominently inside the clinics. Every clinic should have a designated staff in charge of monitoring compliance to the standard operating procedures. Irrespective of the nature of screening or diagnostic evaluation, the basic principles and safety protocols as listed in Table 2 should be adhered to in all settings to minimize SARS-CoV-2 transmission risk. To reduce the clinic load as a result of backlogs, some tasks may be delegated to different cadres of health professionals (task-sharing) or to providers who perform less specialized work (task-shifting). Retired nurses or clinicians and fresh medical or nursing school graduates can also be employed. Such workforce reorganization should be supported by appropriate revision of the regulations and an effective training plan. COVID-free cancer-screening hubs may be created and scaled up in a phased manner depending on the demand and available resources.

**TABLE 2:**
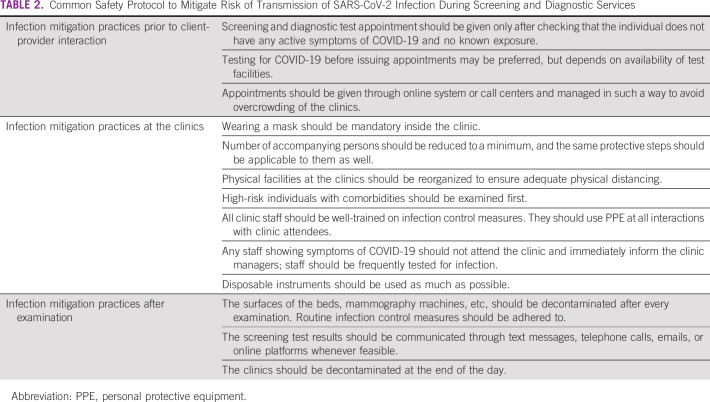
Common Safety Protocol to Mitigate Risk of Transmission of SARS-CoV-2 Infection During Screening and Diagnostic Services

In the following sections, we have discussed the best practice measures that can be adopted to improve efficiency of screening for cervical, breast, and colorectal cancers.

## REORGANIZING CERVICAL CANCER SCREENING SERVICES

The WHO's call to eliminate cervical cancer has motivated efforts across the globe to scale up screening services and implement HPV-based programs. The HPV-negative women won't need any clinic attendance for screening for at least 5 years. HPV-based screening offers a possibility of at-home sample collection by the women themselves, which avoids the use of public transport to clinics and unnecessary exposure within clinics, thereby reducing risk to both health care providers and patients. HPV testing obtained without a gynecologic examination, by women themselves either at home or clinic, provides a reliable result.^17^ The kits for self-collection may be sent by post or distributed by the community health workers.

Majority of the low- and middle-income countries cannot afford HPV tests and have to rely on visual inspection with acetic acid (VIA) test, which requires an examination by a health provider. In such settings, a screen-and-treat approach of immediate treatment of the VIA-positive women will reduce the number of clinic visits by the women and minimize client-provider interactions. We suggest that the current situation actually offers an incentive for the VIA programs to switch to HPV-based screening every 5 or 10 years. Many of the test platforms procured to detect SARS-CoV-2 virus can be used to detect HPV as well.

Depending on the prevailing travel restrictions and load on the services, a policy to triage patients for diagnostic evaluation may be considered. Appropriate triaging of the HPV-positive women can significantly reduce the number of women requiring colposcopy. Triaging HPV-positive women with HPV 16/18 genotyping and cytology (only for those HPV-positive women negative on HPV 16/18) reduced colposcopy referrals by nearly two-third in the screening program in Turkey.^18^ Those with suspected high-grade lesions or cancer on cytology should be given priority appointment for colposcopy over those having low-grade abnormalities.^19^ Outreach VIA, colposcopy, and treatment services, which do not rely on electricity and combine all these interventions in a single visit, could provide a viable alternative to clinic visits in resource-constrained settings.^20^

Women living with HIV have substantially increased risk of cervical cancer. In the current health care landscape, it is important that screening services continue especially for this group of women. The WHO has recommended integrating cervical cancer screening services into antiretroviral therapy programs and screening should be planned at one of the scheduled visits to the antiretroviral therapy clinic.^21^

To minimize number of visits, colposcopically suspected high-grade lesions should be treated immediately without waiting for histopathologic verification. Observational studies have documented the presence of HPV DNA (the type identical to that present in the cervical lesion) in the smoke generated during large loop excision of transformation zone (LLETZ) procedure and transmission of HPV DNA to the operator's nasal cavity.^22,23^ No formal study has yet documented the risk of an operator of getting infected with SARS-CoV-2 because of the aerosols generated during LLETZ procedure. A study on 35 patients infected with SARS-CoV-2 (eight patients were symptomatic) in China demonstrated that none of them had the virus detected in vaginal fluid or cervical specimen.^24^ The British Society of Colposcopy and Cervical Pathology advises to limit the use of diathermy to coagulate the bleeding points after LLETZ (to minimize dispersal of vaporized particles) and liberally use Monsel’s solution for hemostasis.^25^ Thermal ablation and cryotherapy treat the cervical precancerous lesions by tissue desiccation and freezing, respectively, and do not generate any smoke. Nevertheless, the use of PPE is recommended for the provider and assistants while performing colposcopy and treatment of an asymptomatic patient without known COVID status and it should comprise sterile fluid-repellent surgical gloves, eye protection, FFP2 mask, surgical cap, and gown.^26^

## REORGANIZING CRC SCREENING SERVICES

Coordinated efforts are necessary to overcome the COVID-19 challenges and facilitate the resumption of CRC screening programs in a gradual and prioritized manner. Selecting candidates according to individual CRC risk with priority given to high-risk individuals may be considered in a program on the basis of invitation. This process can be coordinated with the screening registry staff or general practitioner who can stratify individuals by risk through phone calls or administration of online questionnaire. In addition, scientific societies should revisit some of their guidelines for patients with inflammatory bowel disease or postpolypectomy endoscopic surveillance to optimize the colonoscopy resources.^27^

Screening and diagnostic services should be reintroduced in COVID-free centers to mitigate risk of infection and encourage population to participate. D'Ovidio et al^28^ conducted a study in Italy to verify the effectiveness and safety in performing selective diagnostic colonoscopies as part of the CRC screening program in a hospital declared as COVID-free hospital. They found that in spite of fewer cases done during lockdown in comparison with data from the same period in 2019, both invasive cancer detection rate (8% *v* 1%; *P* = .002) and high-risk adenoma detection rate (47% *v* 25%; *P* = .001) were higher because of the prioritization of more high-risk cases.

During the pandemic, flexibility in CRC screening methods should be considered. Switching to FIT in programs that use endoscopy-based screening is an example. Moreover, applying an FIT test would allow using the mail system to send and return test kits. Alternatively, the samples can be deposited in drop boxes kept at health facilities. By sending the FIT test kits to the eligible individuals by post, the National Bowel Cancer Screening Program in Australia could maintain reasonably high participation rate to CRC screening even during the worst period of the COVID-19 outbreak.^8^ Quantitative FIT should replace qualitative FIT as it is throughput and reduces the colonoscopy referrals.^29,30^

## REORGANIZING BREAST CANCER SCREENING SERVICES

Differently from cervical and colon cancer screening, breast cancer screening has no alternative for a self-collected screening test. As the screening activities resume while the COVID-19 pandemic is still ongoing, breast imaging and further assessment must be performed using safe practices. On that note, we must balance the need to avoid delays of a breast cancer diagnosis while avoiding infection, which requires careful attention to PPE, physical distancing, and vigilance to maintain these practices; resuming activities should also involve prioritization and implementation of innovative interventions.^31^

Recommendations from different medical associations and experts are proposing that the diagnostic procedures in symptomatic patients should be prioritized over screening procedures. All efforts should be made to avoid delayed diagnosis in situations as clinical suspicion of inflammatory or locally advanced breast cancer, imaging findings of BI-RADS 5 (high priority) or BI-RADS 4 (medium priority), symptomatic women with a new palpable lump or breast thickening that is clinically concerning, and suspicion of breast cancer in a pregnant woman.^31-35^

Annual mammographic screening should be discouraged. One interesting strategy to reduce the number of clinic visits for the women may be a one-stop breast care. Health facilities would offer same-day diagnosis and integrated multidisciplinary care services, which include counseling, mammography, or clinical breast examination followed by appropriate diagnostic evaluation, which includes imprint cytology of breast core biopsy specimens. This model has been tested in both low- and high-resource settings, showing encouraging feasibility and outcomes.^36,37^

## ORGANIZING TREATMENT OF PATIENTS WITH CANCER

The COVID pandemic has significantly disrupted treatment of patients with cancer with patients and the treating oncologists being hesitant to initiate or continue with treatment given the threat of the infection.^38^ Cancer as a comorbidity certainly increases the risk of getting severe complications and dying from COVID.^39^ However, at least two studies have shown that cancer treatment does not increase the threat to life of a patient, even if they get infected during the course of treatment.^40,41^ Strategies that should be considered to ensure timely and adequate treatment of the patients with cancer during various phases of the pandemic are listed in Table 3.

**TABLE 3:**
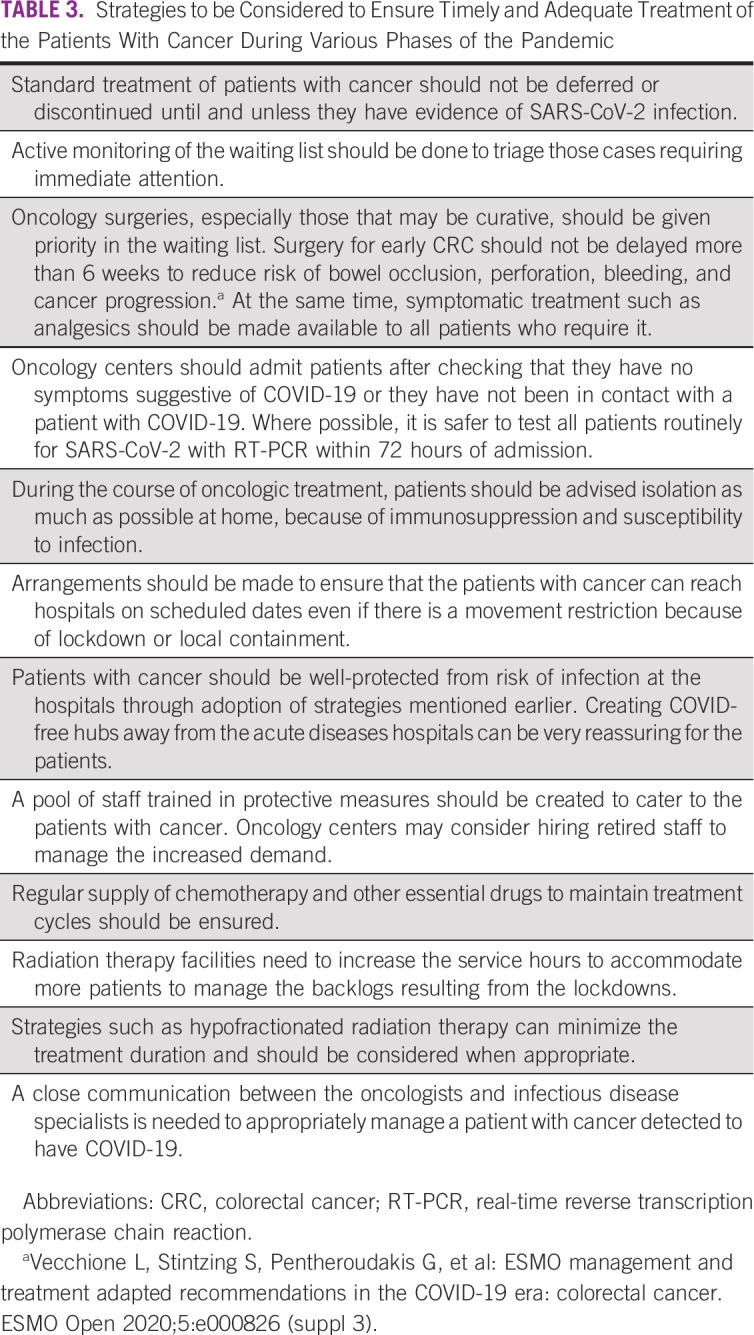
Strategies to be Considered to Ensure Timely and Adequate Treatment of the Patients With Cancer During Various Phases of the Pandemic

## CONTINUATION OF RESEARCH IN CANCER EARLY DETECTION

The COVID-19 pandemic has had a huge impact on clinical research other than those related to the infection and its prophylaxis or treatment.^42^ Many clinical trials were put on hold as there were a lot of uncertainties around the balance of risks and benefits and how best to communicate the same to the prospective participants.^43^ Research evaluating the efficacy and/or implementation of new technologies and approaches in cancer early detection will face major challenges in the near future. IARC planned a multicentric trial to evaluate automated visual examination (artificial intelligence–based recognition of cervical lesions) as a cervical cancer screening tool in India and Thailand.^44^ The study initiation has been delayed by at least 10 months because of less frequent meetings of the ethics committees, suspension of courier services making procurement of consumables and equipment difficult, cessation or slowing down of cancer screening activities, the investigators being preoccupied with COVID-related duties, uncertainties around compliance of the trial participants, and safety concerns of the participants as well as staff because of the increased number of interactions. The biotechnology industries are quite cautious in investing in new innovations in the midst of the economic downturn. Some of the developers of new point-of-care HPV detection tests are investing their energy and resources in new technologies for the detection of SARS-CoV-2 and postponed the development of the HPV tests. The availability of fund for research will shrink significantly with major research-funding agencies already announcing major cuts in cancer research funding.^10^

It is crucial to obtain clear directives and guidance from the national regulatory agencies for continuation of clinical research. To date, the US Food and Drug Administration and European Medicines Agency have published such guidelines.^45,46^ It will be necessary to modify some of the regulatory requirements. Remote consenting through online consultation followed by obtaining a digital signature (e-consent) and follow-up by telephone or video calls in situations not requiring sample collection or assessment by a health provider may become acceptable norms. Advantage should be taken of the enhanced digital literacy and acceptance of app-based COVID-19 symptom–reporting and alert tools to promote self-reporting of outcomes by trial participants using online platforms. The relationship between exposure to the trial-related interactions and subsequent infection with SARS-CoV-2 should be closely monitored, and appropriate actions should be taken if there are any concerns. Implementation research to identify the most pragmatic, acceptable, and cost-effective strategies to continue with cancer screening and treatment services during various phases of the epidemic should be prioritized.

In conclusion, reintegration of individuals to the screening pathway depending on where they left at the time of suspension requires a coordinated approach and involvement of many stakeholders. It is an opportunity to learn from other countries as this has become a global problem and countries are at different stages of adapting cancer screening successfully to the changing health ecosystem. It is also an opportunity to improve the resilience of screenings to threats, which are likely to arise in a volatile and uncertain context. In these unprecedented times, reassessing a cancer screening program could be an opportunity of building back better services. Performing a situation analysis and planning accordingly, prioritizing and keeping focus on evidence-based practices, considering implementation of evidence-based innovations (eg, telehealth and new algorithms), and considering de-implementation of non–evidence-based practices (eg, screening for prostate or liver cancer, screening off-target age population, etc) could be a good start point. The key is to remain flexible and vigilant and periodically assess severity of the outbreak in the population and adapting guidelines to the changing situation.
